# Simultaneous Removal of Cu^2+^, Cd^2+^ and Pb^2+^ by Modified Wheat Straw Biochar from Aqueous Solution: Preparation, Characterization and Adsorption Mechanism

**DOI:** 10.3390/toxics10060316

**Published:** 2022-06-10

**Authors:** Yangyang Wang, Kaixuan Zheng, Zhiqiang Jiao, Wenhao Zhan, Shiji Ge, Shaopeng Ning, Shiyuan Fang, Xinling Ruan

**Affiliations:** 1 National Demonstration Center for Environmental and Planning, College of Geography and Environmental Science, Henan University, Kaifeng 475004, China; wangyangyangxyz@163.com (Y.W.); 104753190175@vip.henu.edu.cn (K.Z.); ZQJiao@henu.edu.cn (Z.J.); GSGe@henu.edu.cn (S.G.); Ningshaopeng@henu.edu.cn (S.N.); 2013040019@henu.edu.cn (S.F.); 2 Key Laboratory of Geospatial Technology for the Middle and Lower Yellow River Regions (Henan University), Ministry of Education, Kaifeng 475004, China; 3 Henan Engineering Research Center for Control & Remediation of Soil Heavy Metal Pollution, Henan University, Kaifeng 475004, China; 4 National Key Laboratory of Human Factors Engineering, China Astronaut Research and Training Center, Beijing 100094, China; zhanwenhao2005@126.com

**Keywords:** adsorption, biochar, mechanisms, metals, modify

## Abstract

As an eco-friendly and efficient adsorbent for removal of potential toxic metals from aqueous solution, biochar has received widespread attention. In the present study, wheat straw biochar (BC) and corresponding modified biochar (HNC) were used to remove Cu^2+^, Cd^2+^ and Pb^2+^ from an aqueous solution. The influence of the environment factors on metals adsorption and adsorption mechanism were discussed in detail. The results showed that the HNC had porous structures and owned ample functional groups (−OH, −COOH and C−N groups) compared with the BC. In the single system, the adsorption capacities of HNC for Cu^2+^, Cd^2+^ and Pb^2+^ at a pH of 5.5 were 18.36, 22.83 and 49.38 mg/g, which were 76.89%, 164.36% and 22.75% higher than that of the BC, respectively. In addition, the adsorption process of Cu^2+^ and Cd^2+^ on BC and HNC fitted to the Langmuir isotherm model and pseudo-second-order kinetics, but the adsorption of Pb^2+^ on BC and HNC fitted to the Langmuir isotherm model and pseudo-first-order kinetics. Adsorption isotherms indicated that the adsorption of Cu^2+^, Cd^2+^ and Pb^2+^ by BC and HNC was a spontaneous endothermic process. The competitive adsorption of mixed metal ions (Cu^2+^, Cd^2+^ and Pb^2+^) revealed that HNC was more preferential to adsorb Cu^2+^ compared with Cd^2+^ and Pb^2+^. Furthermore, Fourier transform infrared spectroscopy and X-ray photoelectron spectroscopy analyses revealed that the main adsorption mechanisms were surface complexation and precipitation, and the adsorbed Cu^2+^, Cd^2+^ and Pb^2+^ on HNC mainly exist as CuO, Cd(OH)_2_, Pb_3_O_4_ and Pb(OH)_2_.

## 1. Introduction

The rapid development of industrial, mining and metallurgical industry has resulted in large amount of metals-containing wastewater being discharged into the natural aquatic environment, especially for Cu^2+^, Cd^2+^ and Pb^2+^, which are common pollutants in the environment [1,2]. These metals are persistent, toxic and biologically accumulating [3,4,5]. When plants absorb nutrients from the polluted environment, these metals can also be accumulated in the organism through the food chain, then causing a long-term threat to the health of humans and ecosystems [6]. Therefore, it is imperative to reduce the discharge of metals into the aquatic environment.

Various methods have been developed to remove metals from the aquatic environment, including adsorption [7], chemical precipitation [8], ion exchange [9], membrane processes and electrolysis reduction [10]. Among these, adsorption has the advantages of a simple operation, strong applicability and low cost, and it is the most widely used method in metal pollution treatment [11]. Fly ash has been used to remove Cu, Mn, Pb, Zn and Cd simultaneously in a previous report, and its adsorption capacity reached 43.78, 17.96, 69.95, 94.43 and 37.49 µg/g, respectively [12]. However, fly ash itself contains various metals [13], which may have a high potential risk of secondary pollution. Fe_3_O_4_@SiO_2_@l-Cysteine functionalized magnetic graphene oxide nanocomposites can remove Cr^6+^, Pb^2+^ and Cd^2+^ efficiently from an aquatic solution, with the maximum adsorption capacity of 476.19, 526.32 and 588.23 mg/g, respectively [14]. However, the complex preparation process and high cost has limited its large-scale application in the treatment of metals-containing wastewater. Therefore, it is necessary to develop a more efficient and low-cost adsorbent to deal with metal pollution in the aquatic environment.

Biochar, as a carbon-rich solid material, can be produced by agricultural wastes, and it has been widely used in the remediation of metals pollution in aquatic environments [15]. Branches of *Robinia pseudoacacia* and durian shells were used to prepare biochar for remediating metals-contaminated water, and the results revealed that their maximum adsorption capacities for Cd^2+^ were 18.18 and 54.11 mg/g, respectively [16]. The adsorption capacity for Cd^2+^ by a wheat straw biochar prepared at 600 °C was 17.92 mg/g [17]. In addition, the wheat straw biochar prepared at 350 and 550 °C just removed less than 3% of the Cd^2+^ when its initial concentration was at 100 mg/L [18]. Hence, most of the biochars obtained by direct pyrolysis of biomass have limited surface functional groups and a relatively low porosity, which affect their adsorption capacity for metals [19]. Therefore, how to improve the adsorption capacity of biochars for metals has become a research hotspot in recent years.

Numerous studies have shown that physical modification by ball-milling and chemical modification by acids, bases, grafting of functional groups and transition metals (e.g.,: Fe, Mn et al.) can change the properties and structure of biochar and improve their metals adsorption capacity [20,21,22,23]. The ball-milling process can improve the metal adsorption capacity by increasing the surface areas of biochar and introducing acidic functional groups to the biochar surface [24,25,26]. The U(VI) adsorption capacity by an HNO_3_-modified wheat straw biochar increased 40 times compared with the unmodified biochar [27]. In addition, the Cd^2+^ adsorption capacity by a sulfhydryl-modified biochar increased by. approximately 300% compared with that of the unmodified biochar [23,28]. However, the high energy consumption, complicated preparation process and harsh preservation conditions (thiol can be oxidized easily) have limited their practical application. In addition, most of previous studies were carried out in a single-metal solution system, and the studies carried out in a multi-metals solution system are relatively few.

We hypothesize that the multiple modifications can significantly improve the metal removal efficiency under both single- and multi-metals solution systems. To test this hypothesis, a wheat straw generated biochar (BC) and corresponding modified biochar (HNC) were used to remediate metals contamination in an aqueous solution. The influence of environmental factors on Cu^2+^, Cd^2+^ and Pb^2+^ adsorption under a monometal and ternary system were comparatively investigated in detail. The potential adsorption mechanism of Cu^2+^, Cd^2+^ and Pb^2+^ on HNC were also analyzed. The results of present study can provide an effective absorbent for the removal of multi-metals in wastewater.

## 2. Materials and Methods

### 2.1. Materials and Reagents

The wheat straw used in the present study was collected from a village in Ruzhou City, Henan Province, China (112°40′54.3″ E, 35°9′34.2″ N). Chemicals and reagents used in the present study were all of analytical grade and purchased from Sinopharm Chemical Reagent Co. Ltd. (Beijing, China), including Cd(NO_3_)_2_∙4H_2_O, Cu(NO_3_)_2_∙3H_2_O, Pb(NO_3_)_2_, NaOH, HNO_3_, KOH and NH_3_(aq). Ultrapure water with a resistivity of 18.25 MΩ cm was adopted in experiments.

### 2.2. Synthesis of BC and HNC

The wheat straw was cleaned successively with tap water and deionized water, then cut into small sections of about 2 cm, dried and pyrolyzed in a tube furnace under limited oxygen (under the protection of N_2_) at 350 °C for 2 h. After naturally cooling to room temperature, the pristine biochar was obtained, grounded, sieved through 100 mesh sizes (0.150 mm) and named as BC. Based on the results of previous studies [29,30], BC was modified successively with KOH (2 mol/L), HNO_3_ (1 mol/L) and ammonia solution (5%, *w*/*w*), then washed repeatedly with deionized water until neutral. After drying, the amino-modified biochar was obtained and named as HNC. The detailed modification process is provided in the Supplementary Materials.

### 2.3. Characterization of BC and HNC

The point of zero charge (pH_pzc_) of BC and HNC was measured according to the method described by Ren et al. [31]. The content of oxygen-containing functional groups on the surface of BC and HNC was determined by Boehem titration [32]. The element (C, H, N, S and O) content of BC and HNC was determined by an elemental analyzer (FLASH 2000HT, Thermo Fisher Scientific, America). The morphology and structure of BC and HNC were observed by scanning electron microscopy (SEM, JSM-7001F, JEOL, Japan). The surface functional groups were obtained via Fourier transform infrared spectroscopy (FT-IR, Vertex 70, Bruker, Chicago, IL, USA) within 4000–400 cm^−1^. The composition and valence states of major elements were acquired via X-ray photoelectron spectroscopy (XPS, AXIS ULTRA, Kratos, Manchester, UK). The surface area and porosity of BC and HNC were measured with a high precision gas/vapor adsorption measurement instrument (BELSORP-max II, Microtrac BEL, Japan). HNC before and after the adsorption of Cd^2+^, Pb^2+^ and Cu^2+^ were analyzed by FT-IR and XPS to investigate the potential adsorption mechanism.

### 2.4. Batch Adsorption Experiments

All adsorption experiments were carried out in a thermostat shaker at 180 rpm/min using a 50 mL flask. HNO_3_ or NaOH (0.1 mol/L) was used to adjust the pH of the solution. Under the monometal system, the influence of the initial pH (1.5, 2.5, 3.5, 4.5, 5.5, 6.5), dosage (0.4, 1.0, 2.0, 4.0, 8.0 g/L), reaction time (0, 5, 10, 15, 20, 30, 40, 60, 90, 120, 240, 360 min), temperature (288.15, 298.15, 308.15 K) and substrate concentration (0, 20, 50, 100, 200, 500, 800, 1000, 1200 mg/L) on Cu^2+^, Cd^2+^ and Pb^2+^ adsorption by both biochars were investigated in detail. The detailed experimental conditions are provided in the Supplementary Materials. Under the ternary system, the adsorption isothermal experiments were carried out with the initial metals concentration of 0–3000 mg/L (the mass ratio of Cu^2+^, Cd^2+^ and Pb^2+^ is 1:1:1), the initial pH of 5.5, the dosage of both biochars of 2 g/L and the temperature of 298.15 K. After 4 h adsorption, the solution samples were collected and used for metals detection. The residual Cu^2+^, Cd^2+^ and Pb^2+^ in all the collected samples were determined by inductively coupled plasma-atomic emission spectrometry (ICP-AES, Thermo fisher ICAP 6200).

The adsorption capacity (qe, mg/g) as well as the removal percentage (R, %) were calculated by the following formula (Equations (1) and (2)):

(1) $$q_{e}=\frac{\left(C_{0}-C_{e}\right)\times V}{M}$$

(2) $$R\%=\frac{C_{0}-C_{e}}{C_{0}}\times 100\%$$

where *C*_0_ and *C_e_* are the initial and equilibrium concentrations of Cu^2+^, Cd^2+^ and Pb^2+^ (mg/L), respectively; *q_e_* is the adsorption amount at equilibrium; *V* is the volume of the solution (L) and *M* is the mass of the adsorbent (g).

To better understand the adsorption mechanism, a Pseudo-first-order (Equation (3)), and Pseudo-second-order models [Equation (4)] were used to fit the adsorption process [33].

(3) $$\ln\left(Q_{e}-Q_{t}\right)=\ln Q_{e}-k_{1}t$$

(4) $$\frac{t}{Q_{t}}=\frac{1}{k_{2}Q_{e}^{2}}+\frac{1}{Q_{e}}t$$

where *Q_e_* and *Q_t_* (mg/g) are the adsorption amount of Cu^2+^, Cd^2+^ and Pb^2+^ at equilibrium and at *t* (min), respectively; *k*_1_ (1/min) and *k*_2_ (g/mg min) are the rate constants of the pseudo-first-order and pseudo-second-order models, respectively.

The adsorption isotherms of Cu^2+^, Cd^2+^ and Pb^2+^ by biochar were fitted by Langmuir (Equation (5), Freundlich (Equation (6)) and Temkin (Equation (7)) equations [33].

(5) $$\frac{C_{e}}{Q_{e}}=\frac{1}{k_{L}q_{max}}+\frac{C_{e}}{q_{max}}$$

(6) $$\ln q_{e}=\ln K_{F}+\frac{1}{n}\ln C_{e}$$

(7) $$q_{e}=\frac{RT}{B}\ln A+\frac{RT}{B}\ln B$$

where *C_e_* refers to the concentration of Cu^2+^, Cd^2+^ and Pb^2+^ at equilibrium (mg/L); qmax is the theoretical maximum adsorption amount (mg/g); *q_e_* is the adsorption amount of Cu^2+^, Cd^2+^ and Pb^2+^ by BC and HNC at equilibrium (mg/g); *K_L_* is the Langmuir constant (L/mg); *K_F_* [mg/g (L/mg)1/n] and 1/n are Freundlich constants. *R* is the universal gas constant (8.314 J/mol K); *T* is the temperature (K) and *A*(L/g) and *B* are the Temkin constants.

### 2.5. Adsorption Mechanism

To further explore the metals adsorption mechanism of HNC, the HNC before and after the adsorption of Cu^2+^, Cd^2+^ and Pb^2+^ (pH 5.5, dosage of 2 g/L for Cu^2+^ and Cd^2+^ and 1 g/L for Pb^2+^, time of 240 min, initial concentration of Cu^2+^, Cd^2+^ and Pb^2+^ of 50 mg/L and temperature of 298.15 K) were analyzed by FT-IR and XPS.

## 3. Results and Discussion

### 3.1. Characterization of Biochar

**Figure 1:**
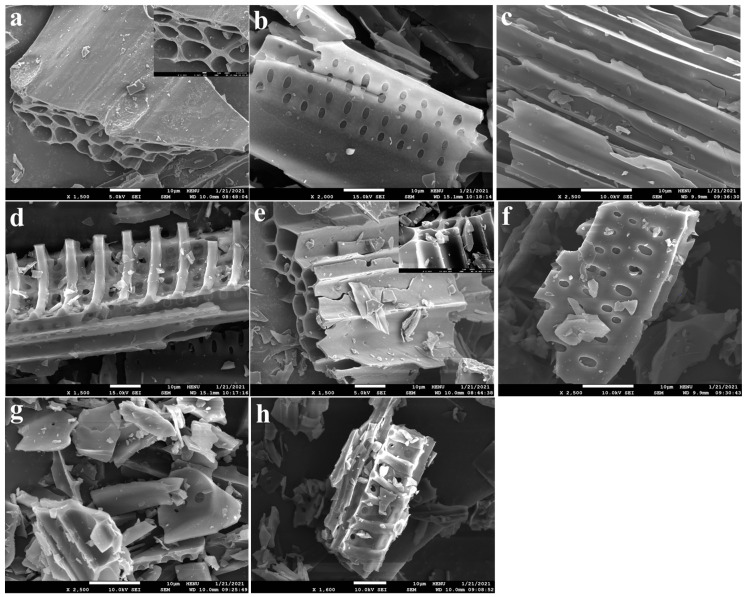
Morphological structure of BC (**a**–**d**) and HNC (**e**–**h**).

The morphological structures of BC and HNC are shown in Figure 1. The structure of BC is tube-shaped, sieve-pored, layered and skeleton-like (Figure 1a–d), which is relatively complete. Conversely, the layered structure was broken (Figure 1e), and the tube-shaped, sieve-pored and skeleton-like structure was collapsed after the modification (Figure 1f–h). In addition, the pore size of HNC was larger than that of the BC, and the average pore diameter increased from 10.20 to 13.46 nm. Compared with BC, the specific surface area, pore volume and micropore volume of NHC all decreased obviously (Table 1). The element analysis indicated that the C content of BC decreased dramatically from 76.29% to 57.55% after the modification. Correspondingly, the content of H, O and N increased from 3.88%, 18.38% and 0.51% to 6.26%, 27.52% and 4.44%, respectively. The atomic ratios of H/C, O/C and (O+N)/C were 0.05, 0.24 and 0.25 for BC, which were increased to 0.11, 0.48 and 0.56, respectively, of that of the HNC. These results indicate that HNC had stronger hydrophilicity and greater polarity, and the aromaticity was weakened [34,35], which may be more conducive for the adsorption of metals [36].

The pH_pzc_ of BC and HNC were 4.77 and 2.69, respectively (Table 1). The decrease of HNC pH_pzc_ (compared with BC) may be favorable for metal cations adsorption due to the enhanced electrostatic attraction [37]. In addition, the modification increased the carboxyl and lactonic groups from 1.251 and 1.206 to 2.320 and 1.336 mmol/g. However, the hydroxyl group decreased correspondingly from 1.975 to 1.670 mmol/g, respectively (Table 1). The FT-IR spectrum of BC and HNC are shown in Figure S1. The spectrum of BC contains peaks at 3417 cm^−1^ (−OH stretching) [38,39], 2921 cm^−1^ (−CH_2_ stretching) [40], 2378 cm^−1^ (C≡N stretching), 1627 cm^−1^ (C=O or −COOH stretching) [41] and 1402 cm^−1^ (C−O or C−N stretching) [42]. The FT-IR spectrum of HNC is similar to that of BC, with some differences in peak intensity and position. The most important changes are the enhanced intensity at 1627 cm^−1^ and 1402 cm^−1^, indicating that the number of −COOH and C−N on the surface of HNC increased. In addition, the decrease of −OH was attributed to the increase of −COOH or C−N. The increase of oxygen- and nitrogen-containing functional groups may enhance the metals removal capacity of HNC through complexing [43].

**Figure 2:**
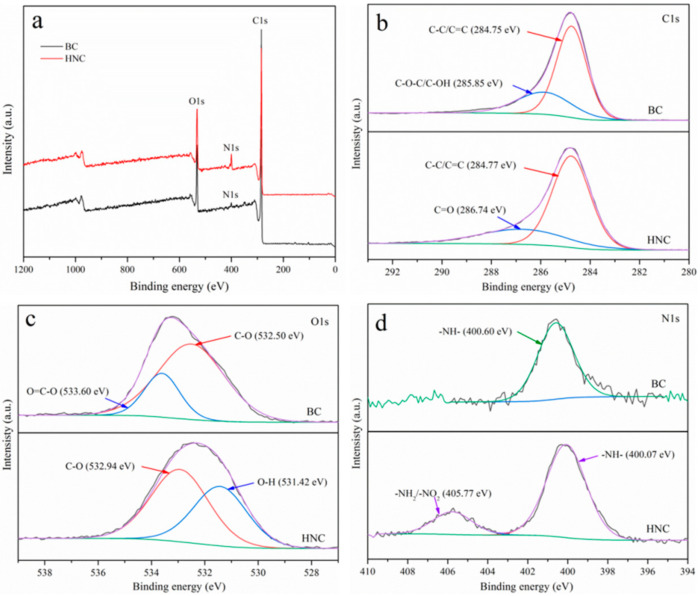
The full XPS spectrum of BC and HNC (**a**); high-resolution XPS spectrum of C1s (**b**), O1s (**c**) and N1s (**d**) of BC and HNC.

As shown in Figure 2a, the peaks that appeared at binding energies of 284.75, 532.5 and 400.60 eV can be assigned to C1s, O1s and N1s, respectively. The C1s (Figure 2b) peak can be divided into four components: 284.75 eV (C−C/C=C) and 285.85 eV (C−O−C/C=OH) in BC and 284.77 eV (C−C/C=C) and 286.74 eV (C=O) in HNC [44]. The O1s spectra (Figure 2c) displayed two peaks: 532.50 eV (C–O) and 533.60 eV (O–C–O) in BC and 531.42 eV (O–H) and 532.94 eV(C–O) in HNC [45]. Moreover, the N1s peaks (Figure 2d) displayed 400.60 eV (−NH−) in BC and 400.07 (−NH−) and 405.77 eV in HNC (−NH_2_/−NO_2_) [46]. The atomic% of N1s increased from 0.68% to 5.78% after the modification, and the new peaks also appeared in the N1s spectrum at about 405.77 eV, which confirmed that aminos were successfully grafted on HNC.

### 3.2. Adsorption of Metals by BC and HNC

#### 3.2.1. Effect of Initial pH

**Figure 3:**
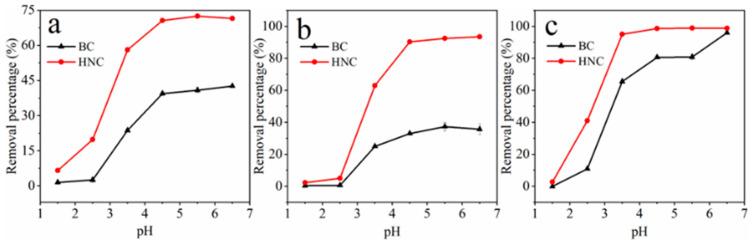
Effect of initial pH on adsorption of Cu^2+^ (**a**), Cd^2+^ (**b**) and Pb^2+^ (**c**) by BC and HNC; dosage of 2 g/L for Cu^2+^ and Cd^2+^ and 1 g/L for Pb^2+^, time of 240 min, initial concentration of Cu^2+^, Cd^2+^ and Pb^2+^ of 50 mg/L, temperature 298.15 K.

The effects of initial pH (1.5–6.5) on the removal of Cu^2+^, Cd^2+^ and Pb^2+^ by BC and HNC are shown in Figure 3. The removal percentage of Cu^2+^, Cd^2+^ and Pb^2+^ was the lowest at the pH of 1.5, which can be attributed to the fact that the initial pH is lower than the pH_pzc_ of BC (pH_pzc_ = 4.77) and HNC (pH_pzc_ = 2.69) (Table 1). With increasing of the initial pH from 1.5 to 4.5, the removal percentage of metals by BC and HNC increased from 1.5% and 6.6% to 39.4% and 70.6% for Cu^2+^, 0.4% and 2.4% to 33.0% and 90.4% for Cd^2+^ and 0% and 2.7% to 80.6% and 98.6% for Pb^2+^, respectively. The change of these removal percentages was nonsignificant when the initial pH increased from 4.5 to 6.5, except for Pb at pH 6.5 by BC. These results demonstrate that the initial pH had a significant impact on metals adsorption by BC and HNC.

In fact, the solution pH can influence the surface charge of the adsorbent and the speciation of metal ions in solution [47]. A lower pH can result in protonation and more positive surface charges of the adsorbent, which can compete (even through electrostatic repulsion) with metal ions and result in a low removal percentage of Cu^2+^, Cd^2+^ and Pb^2+^ by BC and HNC [48]. However, with the initial pH increased from 1.5 to 4.5, the surface charge changed from positive to negative for HNC, and the surface positive charge decreased for BC, both of which increased the removal percentage of these metals by BC and HNC [16]. In general, the initial pH of 5.5 is higher than the pH_pzc_ of BC and HNC, and a higher initial pH could not further enhance the removal percentage of Cu^2+^, Cd^2+^ and Pb^2+^ in solution. Therefore, a pH value of 5.5 was the optimum condition for the removal of Cu^2+^, Cd^2+^ and Pb^2+^ in the aqueous solution.

#### 3.2.2. Effect of Adsorbent Dosage

**Figure 4:**
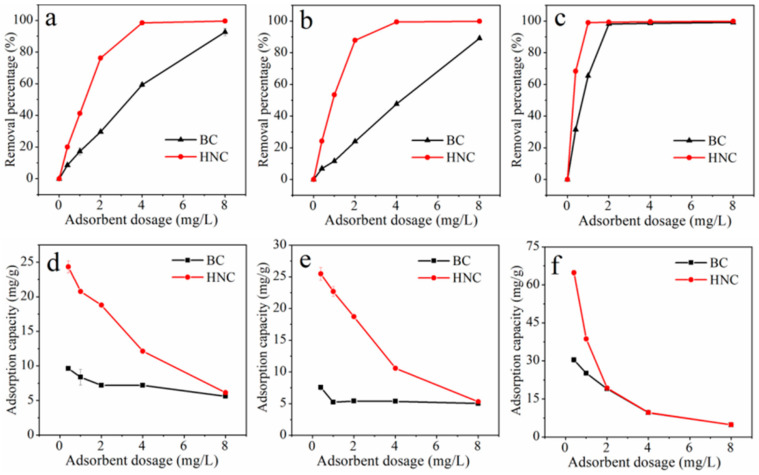
Effect of BC and HNC dosage on the adsorption of Cu^2+^ (**a**,**d**), Cd^2+^ (**b**,**e**) and Pb^2+^ (**c**,**f**) at pH of 5.5, time of 240 min, initial concentration of Cu^2+^, Cd^2+^ and Pb^2+^ of 50 mg/L and temperature of 298.15 K.

The adsorption of Cu^2+^, Cd^2+^ and Pb^2+^ by different dosages of BC and HNC are shown in Figure 4. When their dosage increased from 0.4 g/L to 8.0 g/L, the removal percentage of these metals by BC and HNC increased from 8.56% and 20.12% to 92.75% and 99.59% for Cu^2+^, 6.83% and 24.18% to 89.12% and 99.87% for Cd^2+^ and 31.53% and 68.40% to 99.15% and 99.89% for Pb^2+^ (Figure 4a–c). In general, the removal percentage of Cu^2+^, Cd^2+^ and Pb^2+^ by BC and HNC increased gradually with the increase of their dosage. In contrast, the adsorption capacity decreased from 9.63 mg/g and 24.34 mg/g to 5.63 mg/g and 6.14 mg/g for Cu^2+^, 7.56 mg/g and 25.48 mg/g to 5.03 mg/g and 5.31 mg/g for Cd^2+^ and 30.48 mg/g and 64.84 mg/g to 4.84 mg/g and 4.87 for Pb^2+^ (Figure 4d–f).

Previous studies indicated that the number of the adsorption sites and surface area are positively correlated with the dosage [49], but excessive dosage will result in the agglomeration of the adsorbent, overlapping of the adsorption sites and blocked diffusion of metals, which may be the main reason for the decrease of the adsorption capacity in the present study [50]. In summary, the dosage of 2 g/L for the adsorption of Cu^2+^ and Cd^2+^, and 1 g/L for adsorption of Pb^2+^ was selected for the subsequent adsorption experiments.

#### 3.2.3. Adsorption Kinetics

The adsorption kinetics fitted by the pseudo-first-order and pseudo-second-order models are shown in Figure S3. Figure S3 shows that the adsorption rate at the initial stage was faster, and about 80–90% of these metals were adsorbed within about 1 min, and the adsorption equilibrium was reached at about 20 min. Previous reports indicate that a perfect adsorbent should have a fast adsorption rate and short equilibrium time [51], indicating that HNC may have a good prospect for metals removal in aqueous solutions. In addition, the rapid adsorption at the first stage was mainly controlled by physical diffusion; Cu^2+^, Cd^2+^ and Pb^2+^ occupied the available adsorption sites quickly on the. BC and HNC surface, while chemical adsorption controlled the slow adsorption process until the adsorption equilibrium was reached [52].

The R^2^ of the pseudo-second-order model for Cu^2+^ and Cd^2+^ adsorption by HNC was 0.9880 and 0.9900, respectively, which was higher than that of the pseudo-first-order model (Table 2), indicating that the adsorption of Cu^2+^ and Cd^2+^ by BC and HNC was controlled by chemisorption, such as surface complexation and mineral co-precipitation [53]. In contrast, Pb^2+^ adsorption by HNC fitted better (R^2^ = 0.9992) to the pseudo-first-order kinetics, indicating that the adsorption was controlled by both physical adsorption and chemisorption, especially for the physical adsorption [54]. In addition, the adsorption capacity for these metals by HNC is much higher than that of BC. These results further indicated that the modification significantly increased the metal adsorption capacity of BC.

#### 3.2.4. Adsorption Isotherms

**Figure 5:**
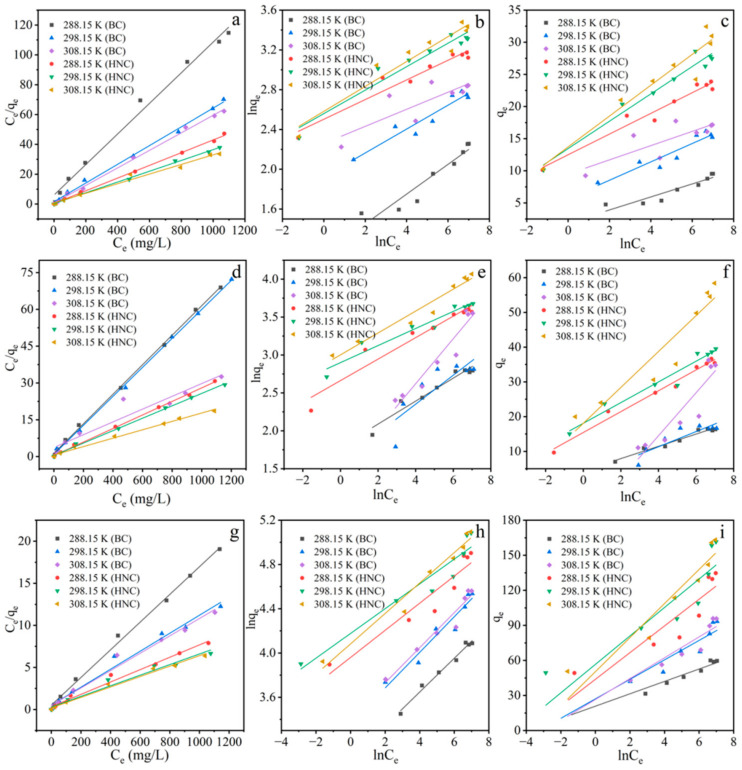
Langmuir, Freundlich and Temkin isotherm fitting of Cu^2+^ (**a**–**c**), Cd^2+^ (**d**–**f**) and Pb^2+^ (**g**–**i**) adsorption by BC and HNC; dosage of 2 g/L (Cu^2+^, Cd^2+^) and 1 g/L (Pb^2+^); pH of 5.5; time of 240 min and temperature of 288.15, 298.15 and 308.15 K.

The fitting parameters of Langmuir, Freundlich and Temkin isotherms are shown in Figure 5 and Table S1. The adsorption of Cu^2+^, Cd^2+^ and Pb^2+^ by BC and HNC fitted the Langmuir models better than the Freundlich and Temkin models (Table S1), indicating that the adsorption of Cu^2+^, Cd^2+^ and Pb^2+^ by both biochars was monolayer chemisorption that occurred on the homogeneous surface [55]. The maximum adsorption capacity of HNC at 288.15, 298.15 and 308.15 K was 23.53, 27.62 and 31.06 mg/g for Cu^2+^; 36.22, 39.56 and 58.48 mg/g for Cd^2+^ and 136.05, 158.73 and 161.29 mg/g for Pb^2+^. The adsorption capacity of Cu^2+^, Cd^2+^ and Pb^2+^ by BC and HNC increased gradually with the increasing temperature. This result indicates that the adsorption of these metals by BC and HNC was an endothermal reaction, which is similar to the previous reports [56,57].

#### 3.2.5. Competitive Adsorption

**Figure 6:**
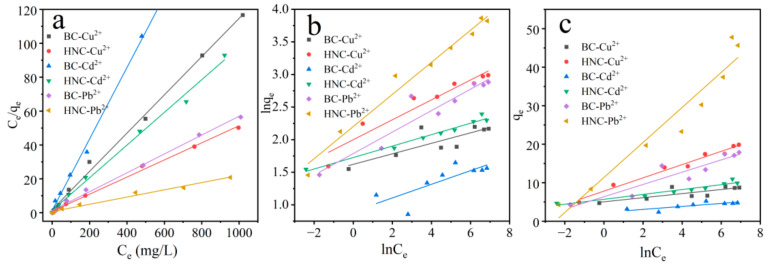
Equilibrium adsorption isotherms of metals by BC and HNC in a ternary system; dosage of 2 g/L, pH of 5.5, time of 240 min and temperature of 298.15 K (Langmuir (**a**), Freundlich (**b**) and Temkin (**c**)).

Langmuir, Freundlich and Temkin isotherms were used to describe the Cu^2+^, Cd^2+^ and Pb^2+^ adsorption process by BC and HNC in a ternary system (Figure 6 and Table 3). As shown in Table 3, the adsorption of Cu^2+^, Cd^2+^ and Pb^2+^ in the ternary system by BC and HNC is similar with that of the monometal system, which fitted better with the Langmuir models than the Freundlich and Temkin. The maximum adsorption capacities (mg/g) for these metals under a ternary system followed the following order: Pb^2+^ (17.95) > Cu^2+^ (8.86) > Cd^2+^ (4.74) by BC, and Pb^2+^ (46.84) > Cu^2+^ (19.79) > Cd^2+^ (10.37) by HNC. The maximum adsorption capacities of these metals by BC and HNC under a ternary system were much lower than those under a monometal system, which might be attributed to the limited adsorption site on both biochars [2]. In addition, compared with the monometal system, the decrease of the maximum adsorption capacity of Cd^2+^ was the most, followed by Pb^2+^ and Cu^2+^. This result may mean that Cd^2+^ and Pb^2+^ could be exchanged and substituted by other metals easily compared with Cu^2+^. Furthermore, HNC showed a higher affinity toward metals compared with BC, which further verified that HNC has a stronger adsorption capacity for metals.

### 3.3. Potential Adsorption Mechanisms

**Figure 7:**
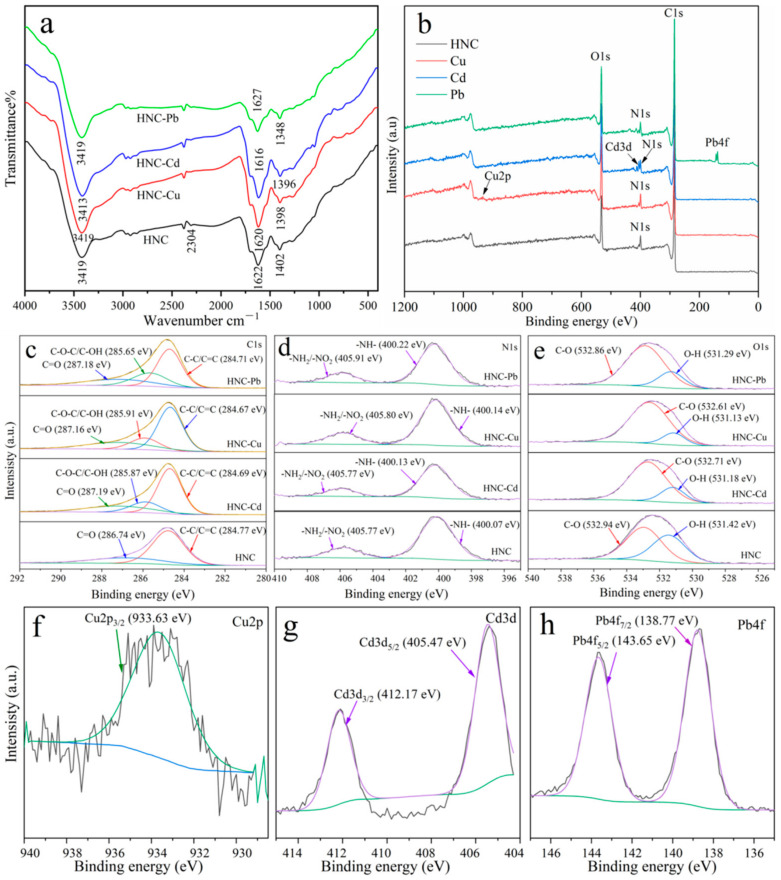
Adsorption mechanism for Cu^2+^, Cd^2+^ and Pb^2+^ by HNC. FT-IR spectra (**a**) and full XPS spectrum (**b**) of HNC before and after the adsorption of Cu, Cd and Pb; high-resolution XPS spectrum of C1s (**c**), O1s (**d**) and N1s (**e**) of HNC before and after the adsorption of Cu, Cd and Pb; high-resolution XPS spectrum of Cu (**f**), Cd (**g**) and Pb (**h**) after the adsorption by HNC.

Fourier transform infrared spectroscopy was used to characterize the adsorption mechanisms of Cu^2+^, Cd^2+^ and Pb^2+^ on HNC (Figure 7a). After the adsorption of Cu^2+^, Cd^2+^ and Pb^2+^, the peaks at 3419 cm^−1^ (−OH group) and 1402 cm^−1^ (C−O or C−N group) shifted slightly. Meanwhile, the area of these peaks decreased obviously, demonstrating that these groups participate in the metal adsorption [38,39,42]. At the same time, the peak at 2304 cm^−1^ disappeared, which confirmed that the O=C=O group also participates in the adsorption of metals [58]. Furthermore, the peak at 1622 cm^−1^ (C=O or −COOH) was shifted and sharpened after the adsorption of Cu^2+^ and Cd^2+^ but weakened after the adsorption of Pb^2+^, which indicates that aromatic C=O and −COOH also participate in the metals adsorption [33]. The above results verified that multiple functional groups (−OH, C−O or C−N, C=O or −COOH and O=C=O) participated in the adsorption of metals through electrostatic interaction and surface complexation.

The X-ray photoelectron spectroscopy analysis indicated that new peaks (Cu 2p, Cd 3d and Pb 4f) appeared after the adsorption of Cu^2+^, Cd^2+^ and Pb^2+^ (Figure 7b). The binding energy of C1s (Figure 7c) at 284.77 and 286.74 eV can be assigned to C−C/C=C and C=O, respectively. After the adsorption of Cu^2+^, Cd^2+^ and Pb^2+^, the new peaks representing C−O−C/C−OH appeared in the C 1s spectrum at about 285.91, 285.87 and 285.65 eV, respectively [59]. Meanwhile, the peak area of C−C/C=C decreased from 72.37% to 57.84%, 60.10% and 51.34% after the adsorption of Cu^2+^, Cd^2+^ and Pb^2+^, respectively, indicating that C−C/C=C played an important role in the adsorption of metals. The N1s (Figure 7d)) spectra of HNC before the metal adsorption can be divided into 400.07 (−NH−) and 505.77 eV (-NH_2_/-NO_2_), while it just shifted slightly after the adsorption of Cu^2+^, Cd^2+^ and Pb^2+^, indicating that −NH− and -NH_2_/-NO_2_ played a small role in the metal adsorption process. After the adsorption of Cu^2+^, Cd^2+^ and Pb^2+^, the proportion of C−O (Figure 7e) significantly increased from 56.70% to 85.90%, 80.20% and 78.94%, respectively. At the same time, the proportion of O−H decreased from 43.3% to 14.1%, 19.8% and 21.06%, respectively. These results imply that the adsorption of metals is accompanied by the breaking of hydrogen bonds [59].

To further identify the occurrence statue of Cu^2+^, Cd^2+^ and Pb^2+^ after the adsorption by HNC, the Cu 2p, Cd 3d and Pb 4f spectra were divided into different components, and the results are shown in Figure 6f–h. The spectra of Cu 2p (Figure 7f) exhibited that the peaks at 933.63 eV (Cu_2p_ 3/2) are in good agreement with that of CuO [60]. The spectra of Cd 3d (Figure 7g) displayed two peaks at 405.47 eV (Cd3d_5/2_) and 412.17 eV (Cd3d_3/2_), which are assigned to the Cd(OH)_2_ (64.22%) and CdCO_3_(35.78%) [61]. Simultaneously, the spectra of Pb 4f (Figure 7h) could be divided into two peaks at 143.65 eV (Pb4f_5/2_, 44.92%) and 138.77 eV (Pb4f_7/2_, 55.08%)) and assigned to Pb_3_O_4_ and Pb(OH)_2_, respectively [62]. These results demonstrate that surface precipitation is the main mechanism for Cu^2+^, Cd^2+^ and Pb^2+^ sorption by HNC.

## 4. Conclusions

In conclusion, the modification dramatically changed the physical and chemical characteristics of BC. HNC had a higher adsorption capacity for Cu^2+^, Cd^2+^ and Pb^2+^ compared with BC. The chemisorption controlled the overall adsorption for Cu^2+^ and Cd^2+^ by BC and HNC, and both physical adsorption and chemisorption controlled for Pb^2+^ adsorption by BC and HNC. The Langmuir model could precisely describe the adsorption isotherm. In addition, HNC was more preferential to adsorb Cu^2+^ in the ternary system compared with Pb^2+^ and Cd^2+^. The predominant metals sorption mechanism by HNC was the surface complexation and precipitation. These results indicate that HNC is a potential adsorbent for the remediation of metals contamination in aqueous environments.

## Figures and Tables

**Table 1:** Physiochemical properties of BC and HNC.

	BC	HNC
pH_pzc_	4.77	2.69
carboxyl group (mmol/g)	1.251	2.320
lactonic group (mmol/g)	1.206	1.336
hydroxyl group (mmol/g)	1.975	1.670
C [%]	76.29	57.55
H [%]	3.88	6.26
O [%]	18.38	27.52
N [%]	0.51	4.44
H/C	0.05	0.11
O/C	0.24	0.48
(O+N)/C	0.25	0.56
BET surface area (m^2^/g)	3.1764	2.7033
BJH pore volume (cm^3^/g)	0.012958	0.010247
Average diameter of pores (nm)	10.198	13.464
Micropore volume (cm^3^/g)	0.0007243	0.0004308

**Table 2:** Kinetic parameters for the adsorption of Cu^2+^, Cd^2+^ and Pb^2+^ by BC and HNC.

Models	Parameters	Values					
		Cu^2+^		Cd^2+^		Pb^2+^	
		BC	HNC	BC	HNC	BC	HNC
Pseudo-first-order	qe,exp (mg/g)	8.104	18.27	7.615	22.63	37.80	49.74
	qe,cal (mg/g)	7.784	17.20	7.219	21.75	34.00	49.05
	k_1_ (1/min)	1.624	2.382	2.401	2.629	1.458	2.444
	R^2^	0.9637	0.979	0.9777	0.9828	0.8976	0.9992
Pseudo-second-order	qe,cal (mg/g)	7.930	17.41	7.298	21.99	33.20	48.67
	k_2_ (g/mg min)	0.457	0.421	1.070	0.392	1.544	3.922
	R^2^	0.9842	0.9880	0.9842	0.9900	0.8609	0.9919

**Table 3:** Isotherm parameters of Langmuir, Freundlich and Temkin models for Cu^2+^_,_ Cd^2+^ and Pb^2+^ by BC and HNC in a ternary system.

Adsorption Isotherm	Parameter	BC			HNC		
		Cu^2+^	Cd^2+^	Pb^2+^	Cu^2+^	Cd^2+^	Pb^2+^
Langmuir	qmax (mg·g^−1^)	8.86	4.74	17.95	19.79	10.37	46.84
	KL (L·mg^−1^)	0.061	0.146	0.043	0.059	0.071	0.029
	R^2^	0.9957	0.9972	0.9958	0.9964	0.9940	0.9836
Freundlich	KF (mg·g^−1^)	5.06	2.50	5.97	7.39	5.62	9.04
	1/n	0.081	0.098	0.165	0.153	0.088	0.246
	R^2^	0.6348	0.5539	0.8774	0.9119	0.9701	0.9739
	A	13,189.7	382.7	53.9	93.9	8081.4	12.2
Temkin	B	4659.7	6407.5	1554.5	1439.7	3912.8	545.4
	R^2^	0.5737	0.6011	0.8432	0.9747	0.9098	0.9221

## Data Availability

Not applicable.

## Supplementary Materials

The following supporting information can be downloaded at: https://www.mdpi.com/article/10.3390/toxics10060316/s1. Figure S1: FT-IR spectra of BC and HNC; Figure S2: Pseudo-first-order kinetic and pseudo-second-order kinetic fitting of Cu^2+^ (a), Cd^2+^ (b) and Pb^2+^ (c) adsorption by BC and HNC; dosage of 2 g/L for Cu^2+^ and Cd^2+^ and 1 g/L for Pb^2+^; pH of 5.5; initial concentration of Cu^2+^, Cd^2+^ and Pb^2+^ of 50 mg/L and temperature of 298.15 K; Table S1: Isotherm parameters for the adsorption of Cu^2+^, Cd^2+^ and Pb^2+^ by BC and HNC.

## References

[1] Thasneema K.K., Dipin T., Thayyil M.S., Sahu P.K., Messali M., Rosalin T., Elyas K.K., Saharuba P.M., Anjitha T., Ben Hadda T. (2021). Removal of toxic heavy metals, phenolic compounds and textile dyes from industrial waste water using phosphonium based ionic liquids. J. Mol. Liq..

[2] Liu J., Hu C., Huang Q. (2019). Adsorption of Cu^2+^, Pb^2+^, and Cd^2+^ onto oiltea shell from water. Bioresour. Technol..

[3] Irshad M., Shakoor M., Ali S., Nawaz R., Rizwan M. (2019). Synthesis and application of titanium dioxide nanoparticles for removal of Cadmium from wastewater: Kinetic and equilibrium study. Water Air Soil Pollut..

[4] Yang L., Ren Q., Zheng K., Jiao Z., Ruan X., Wang Y. (2022). Migration of heavy metals in the soil-grape system and potential health risk assessment. Sci. Total Environ..

[5] Yang L., Ren Q., Ge S., Jiao Z., Zhan W., Hou R., Ruan X., Pan Y., Wang Y. (2022). Metal(loid)s spatial distribution, accumulation and potential health risk assessment in soil-wheat systems near a Pb/Zn smelter in Henan Province, central China. Int. J. Environ. Res. Pub. Health..

[6] Zheng H., Ren Q., Zheng K., Qin Z., Wang Y., Wang Y. (2022). Spatial distribution and risk assessment of metal(loid)s in marine sediments in the Arctic Ocean and Bering Sea. Mar. Pollut. Bull..

[7] Wang Y., Liu H., Wang S., Li X., Wang X., Jia Y. (2020). Simultaneous removal and oxidation of arsenic from water by delta-MnO_2_ modified activated carbon. J. Environ. Sci..

[8] Matlock M.M., Howerton B.S., Atwood D.A. (2002). Chemical precipitation of lead from lead battery recycling plant wastewater. Ind. Eng. Chem. Res..

[9] Ajmal M., Rao R., Anwar S., Ahmad J., Ahmad R. (2003). Adsorption studies on rice husk: Removal and recovery of Cd(II) from wastewater. Bioresour. Technol..

[10] Abu Qdais H., Moussa H. (2004). Removal of heavy metals from wastewater by membrane processes: A comparative study. Desalination.

[11] Li Z.H., Xing B., Ding Y., Li Y., Wang S. (2020). A high-performance biochar produced from bamboo pyrolysis with in-situ nitrogen doping and activation for adsorption of phenol and methylene blue. Chin. J. Chem. Eng..

[12] Mohan S.R., Gandhimathi R. (2009). Removal of heavy metal ions from municipal solid waste leachate using coal fly ash as an adsorbent. J. Hazard. Mater..

[13] Sahoo P.K., Kim K., Powell M.A., Equeenuddin S. (2016). Recovery of metals and other beneficial products from coal fly ash: A sustainable approach for fly ash management. Int. J. Coal. Sci. Technol..

[14] Ghorbani M., Ariavand S., Aghamohammadhasan M., Seyedin O. (2021). Synthesis and optimization of a green and efficient sorbent for removal of three heavy metal ions from wastewater samples: Kinetic, thermodynamic, and isotherm studies. J. Iran. Chem. Soc..

[15] Wang Y., Liu R.H. (2018). H_2_O treatment enhanced the heavy metals removal by manure biochar in aqueous solutions. Sci. Total Environ..

[16] Yang T.T., Xu Y.M., Huang Q.Q., Sun Y.B., Liang X.F., Wang L., Qin X., Zhao L.J. (2021). Adsorption characteristics and the removal mechanism of two novel Fe-Zn composite modified biochar for Cd(II) in water. Bioresour. Technol..

[17] Bogusz A., Nowak K., Stefaniuk M., Dobrowolski R., Oleszczuk P. (2017). Synthesis of biochar from residues after biogas production with respect to cadmium and nickel removal from wastewater. J. Environ. Manag..

[18] Wang Y., Liu R.H. (2017). Comparison of characteristics of twenty-one types of biochar and their ability to remove multi-heavy metals and methylene blue in solution. Fuel Process. Technol..

[19] Faheem, Yu H.X., Liu J., Shen J.Y., Sun X.Y., Li J.S., Wang L.J. (2016). Preparation of MnOx-loaded biochar for Pb^2+^ removal: Adsorption performance and possible mechanism. J. Taiwan Inst. Chem. Eng..

[20] Li B., Yang L., Wang C.Q., Zhang Q.P., Liu Q.C., Li Y.D., Xiao R. (2017). Adsorption of Cd(II) from aqueous solutions by rape straw biochar derived from different modification processes. Chemosphere.

[21] Sizmur T., Fresno T., Akgul G., Frost H., Moreno-Jimenez E. (2017). Biochar modification to enhance sorption of inorganics from water. Bioresour. Technol..

[22] Chen D., Wang X., Wang X., Feng K., Su J., Dong J.N. (2020). The mechanism of cadmium sorption by sulphur-modified wheat straw biochar and its application cadmium-contaminated soil. Sci. Total Environ..

[23] Fan J.J., Cai C., Chi H.F., Reid B.J., Coulon F., Zhang Y.C., Hou Y.W. (2020). Remediation of cadmium and lead polluted soil using thiol-modified biochar. J. Hazard. Mater..

[24] Li F., Wan Y., Chen J., Hu X., Tsang D., Wang H., Gao B. (2020). Novel ball-milled biochar-vermiculite nanocomposites effectively adsorb aqueous As(V). Chemosphere.

[25] Lyu H.H., Gao B., He F., Zimmerman A.R., Ding C., Huang H., Tang J.C. (2018). Effects of ball milling on the physicochemical and sorptive properties of biochar: Experimental observations and governing mechanisms. Environ. Pollut..

[26] Wang B., Gao B., Wan Y.S. (2018). Entrapment of ball-milled biochar in Ca-alginate beads for the removal of aqueous Cd(II). J. Ind. Eng. Chem..

[27] Jin J., Li S., Peng X., Liu W., Zhang C.L., Yang Y., Han L., Du Z.W., Sun K., Wang X. (2018). HNO_3_ modified biochars for uranium (VI) removal from aqueous solution. Bioresour. Technol..

[28] Xiong J., Zhou M.G., Qu C.C., Yu D., Chen H.C., Wang M.X., Tan W.F. (2021). Quantitative analysis of Pb adsorption on sulfhydryl-modified biochar. Biochar.

[29] Wu W., Li J., Niazi N., Müller K., Chu Y., Zhang L., Yuan G., Lu K., Song Z., Wang H. (2016). Influence of pyrolysis temperature on lead immobilization by chemically modified coconut fiber-derived biochars in aqueous environments. Environ. Sci. Pollut. Res..

[30] Xu L., Liu Y., Wang J., Tang Y., Zhang Z. (2021). Selective adsorption of Pb^2+^ and Cu^2+^ on amino-modified attapulgite: Kinetic, thermal dynamic and DFT studies. J. Hazard. Mater..

[31] Ren Z., Zhang G., Chen P.J. (2011). Adsorptive removal of arsenic from water by an iron–zirconium binary oxide adsorbent. J. Colloid Interface Sci..

[32] Boehm H.P., Voll M. (1968). Studies on basic surface oxides of carbon. Carbon.

[33] Chen Y., Li M., Li Y., Liu Y., Chen Y., Li H., Li L., Xu F., Jiang H., Chen L. (2021). Hydroxyapatite modified sludge-based biochar for the adsorption of Cu^2+^ and Cd^2+^: Adsorption behavior and mechanisms. Bioresour. Technol..

[34] Ahmad M., Lee S., Dou X., Mohan D., Sung J., Yang J., Ok Y.S. (2012). Effects of pyrolysis temperature on soybean stover- and peanut shell-derived biochar properties and TCE adsorption in water. Bioresour. Technol..

[35] Al-Wabel M., Al-Omran A., El-Naggar A., Nadeem M., Usman A. (2013). Pyrolysis temperature induced changes in characteristics and chemical composition of biochar produced from conocarpus wastes. Bioresour. Technol..

[36] Qiu B.B., Tao X.D., Wang H., Li W.K., Ding X., Chu H.Q. (2021). Biochar as a low-cost adsorbent for aqueous heavy metal removal: A review. J. Anal. Appl. Pyrol..

[37] Yu Y., Zhang C., Yang L., Chen J. (2017). Cerium oxide modified activated carbon as an efficient and effective adsorbent for rapid uptake of arsenate and arsenite: Material development and study of performance and mechanisms. Chem. Eng. J..

[38] Xue Y.J., Wang C., Hu Z.H., Zhou Y., Xiao Y., Wang T. (2019). Pyrolysis of sewage sludge by electromagnetic induction: Biochar properties and application in adsorption removal of Pb(II), Cd(II) from aqueous solution. Waste Manag..

[39] Yang Y., Chen N., Feng C., Li M., Gao Y. (2018). Chromium removal using a magnetic corncob biochar/polypyrrole composite by adsorption combined with reduction: Reaction pathway and contribution degree. Colloid Surf. A.

[40] Chen R., Cheng Y., Wang P., Wang Q., Wan S., Huang S., Su R., Song Y., Wang Y. (2021). Enhanced removal of Co(II) and Ni(II) from high-salinity aqueous solution using reductive self-assembly of three-dimensional magnetic fungal hyphal/graphene oxide nanofibers. Sci. Total Environ..

[41] Chen Y.N., Zeng Z.P., Li Y.P., Liu Y.H., Chen Y.R., Wu Y.X., Zhang J.C., Li H., Xu R., Wang S. (2020). Glucose enhanced the oxidation performance of iron-manganese binary oxides: Structure and mechanism of removing tetracycline. J. Colloid Interf. Sci..

[42] Tsai W., Liu S., Chen H., Chang Y., Tsai Y. (2012). Textural and chemical properties of swine-manure-derived biochar pertinent to its potential use as a soil amendment. Chemosphere.

[43] Yang X.D., Wan Y.S., Zheng Y.L., He F., Yu Z.B., Huang J., Wang H.L., Ok Y.S., Jiang Y.S., Gao B. (2019). Surface functional groups of carbon-based adsorbents and their roles in the removal of heavy metals from aqueous solutions: A critical review. Chem. Eng. J..

[44] Liu L., Fan S.S. (2018). Removal of cadmium in aqueous solution using wheat straw biochar: Effect of minerals and mechanism. Environ. Sci. Pollut. Res..

[45] Lv Y.C., Niu Z.Y., Chen Y.C., Hu Y.Y. (2017). Bacterial effects and interfacial inactivation mechanism of nZVI/Pd on Pseudomonas putida strain. Water Res..

[46] Chen R., Cheng Y., Wang P., Wang Y., Wang Q., Yang Z., Tang C., Xiang S., Luo S., Huang S. (2021). Facile synthesis of a sandwiched Ti_3_C_2_T_x_ MXene/nZVI/fungal hypha nanofiber hybrid membrane for enhanced removal of Be(II) from Be(NH_2_)_2_ complexing solutions. Chem. Eng. J..

[47] Pehlivan E., Yanik B.H., Ahmetli G., Pehlivan M. (2008). Equilibrium isotherm studies for the uptake of cadmium and lead ions onto sugar beet pulp. Bioresour. Technol..

[48] Zhang P.Z., Zhang X.X., Yuan X.R., Xie R.Y., Han L.J. (2021). Characteristics, adsorption behaviors, Cu(II) adsorption mechanisms by cow manure biochar derived at various pyrolysis temperatures. Bioresour. Technol..

[49] Nair V., Panigrahy A., Vinu R. (2014). Development of novel chitosan-lignin composites for adsorption of dyes and metal ions from wastewater. Chem. Eng. J..

[50] Rahmani A., Mousavi H.Z., Fazli M. (2010). Effect of nanostructure alumina on adsorption of heavy metals. Desalination.

[51] Cai C., Zhao M., Yu Z., Rong H., Zhang C.S. (2019). Utilization of nanomaterials for in-situ remediation of heavy metal(loid) contaminated sediments: A review. Sci. Total Environ..

[52] Wang H., Wang X.J., Ma J.X., Xia P., Zhao J.F. (2017). Removal of cadmium (II) from aqueous solution: A comparative study of raw attapulgite clay and a reusable waste-struvite/attapulgite obtained from nutrient-rich wastewater. J. Hazard. Mater..

[53] Ali R.M., Hamad H.A., Hussein M.M., Malash G.F. (2016). Potential of using green adsorbent of heavy metal removal from aqueous solutions: Adsorption kinetics, isotherm, thermodynamic, mechanism and economic analysis. Ecol. Eng..

[54] Beesley L., Inneh O., Norton G., Moreno-Jimenez E., Pardo T., Clemente R., Dawson J. (2014). Assessing the influence of compost and biochar amendments on the mobility and toxicity of metals and arsenic in a naturally contaminated mine soil. Environ. Pollut..

[55] Trakal L., Veselska V., Safarik I., Vitkova M., Cihalova S., Komarek M. (2016). Lead and cadmium sorption mechanisms on magnetically modified biochars. Bioresour. Technol..

[56] Tan X.F., Liu S.B., Liu Y.G., Gu Y.L., Zeng G.M., Cai X.X., Yan Z.L., Yang C.P., Hu X.J., Chen B. (2016). One-pot synthesis of carbon supported calcined-Mg/Al layered double hydroxides for antibiotic removal by slow pyrolysis of biomass waste. Sci. Rep..

[57] Wang H., Huang F., Zhao Z.L., Wu R.R., Xu W.X., Wang P., Xiao R.B. (2021). High-efficiency removal capacities and quantitative adsorption mechanisms of Cd^2+^ by thermally modified biochars derived from different feedstocks. Chemosphere.

[58] Choudhary M., Kumar R., Neogi S. (2020). Activated biochar derived from *Opuntia ficus-indica* for the efficient adsorption of malachite green dye, Cu^2+^ and Ni^2+^ from water. J. Hazard. Mater..

[59] He S.R., Li Y.T., Weng L.P., Wang J.J., He J.X., Liu Y.L., Zhang K., Wu Q.H., Zhang Y.L., Zhang Z. (2018). Competitive adsorption of Cd^2+^, Pb^2+^ and Ni^2+^ onto Fe^3+^-modified argillaceous limestone: Influence of pH, ionic strength and natural organic matters. Sci. Total Environ..

[60] Zu Y., Guo Z.S., Zheng J., Hui Y., Wang S.H., Qin Y.C., Zhang L., Liu H.H., Gao X.H., Song L.J. (2020). Investigation of Cu(I)-Y zeolites with different Cu/Al ratios towards the ultra-deep adsorption desulfurization: Discrimination and role of the specific adsorption active sites. Chem. Eng. J..

[61] Huang Q.Q., Chen Y., Yu H., Yan L., Zhang J.H., Wang B., Du B., Xing L. (2018). Magnetic graphene oxide/MgAl-layered double hydroxide nanocomposite: One-pot solvothermal synthesis, adsorption performance and mechanisms for Pb^2+^, Cd^2+^, and Cu^2+^. Chem. Eng. J..

[62] Sharma A.S., Biswas K., Basu B. (2013). Fine scale characterization of surface/subsurface and nanosized debris particles on worn Cu-10% Pb nanocomposites. J. Nanopart. Res..

