# Supplementary material for: Simultaneous Removal of Cu2+, Cd2+ and Pb2+ by Modified Wheat Straw Biochar from Aqueous Solution: Preparation, Characterization and Adsorption Mechanism
Source: Toxics. 2022 Jun 10;10(6):316. doi: 10.3390/toxics10060316 (PMC9231304; doi:10.3390/toxics10060316)
Supplement: Supplementary file 1 [file toxics-10-00316-s001.zip › toxics-1753907-supplementary.pdf]

Supplementary Materials

1. The detailed modification process of HNC

20 g of BC was stirred in 2 mol/L KOH solution [1:10 (g/mL)] in a shaking bath at 80 ºC and 220 rpm for 4 h, and then repeatedly washed with deionized water to neutral. Afterwards, 1 mol/L HNO_3 [1:10 (g/mL)] was added, stirred in a shaking bath at 80 ºC and 220 rpm for 4 h, then washed repeatedly with deionized water to neutral. Finally, ammonia solution 5% (w/w) (1:10 (g/mL)) was added, stirred at 50 ºC and at 220 rpm for 4h, and then washed repeatedly with deionized water until neutral.

2. Experimental conditions of the batch adsorption experiments under the monometal system

Effect of initial pH: initial pH=1.5, 2.5, 3.5, 4.5, 5.5 and 6.5, the dosages at 2 g/L for Cu^{2+} and Cd^{2+}, and 1 g/L for Pb^{2+}, reaction time 240 min, temperature 298.15 K, substrate concentration 50 mg/L

Effect of BC and HNC dosage: initial pH=5.5, the dosages 0.4, 1.0, 2.0, 4.0, 8.0 g/L, reaction time 240 min, temperature 298.15 K, substrate concentration 50 mg/L

Adsorption kinetics: initial pH=5.5, the dosages at 2 g/L for Cu^{2+} and Cd^{2+}, and 1 g/L for Pb^{2+}, reaction time 0, 5, 10, 15, 20, 30, 40, 60, 90, 120, 240 and 360 min, temperature 298.15 K, substrate concentration 50 mg/L

Adsorption isotherms: initial pH=5.5, the dosages at 2g/L for Cu^{2+} and Cd^{2+}, and 1g/L for Pb^{2+}, reaction time 240 min, temperature 288.15, 298.15 and 308.15K, substrate concentration 0, 20, 50, 100, 200, 500, 800, 1000, 1200 mg/L

Table S1 Isotherm parameters for the adsorption of Cu^{2+}, Cd^{2+} and Pb^{2+} by BC and HNC

Temperature (K)	Adsorption isotherm	Parameter	Cu^{2+}		Cd^{2+}		Pb^{2+}	
			BC	HNC	BC	HNC	BC	HNC
288.15	Langmuir	q_{max} (mg/g)	9.76	23.53	16.63	36.22	60.79	136.05
		K_L (L/mg)	0.017	0.104	0.045	0.071	0.032	0.023
		R^2	0.9911	0.9986	0.9989	0.9988	0.9965	0.9846
	Freundlich	K_F(mg/g (L/mg)$^{1/n}$)	3.16	12.22	5.96	14.32	20.86	52.46
		$1/n$	0.150	0.099	0.152	0.142	0.153	0.123
		R^2	0.9022	0.9452	0.9447	0.9328	0.9831	0.9026
	Temkin	A	6.4	2344	12.0	170.2	50.8	46.3
		B	2367	1488	1358	798.2	451.9	209.7
		R^2	0.8528	0.9517	0.9573	0.9804	0.9537	0.8644
298.15	Langmuir	q_{max} (mg/g)	15.95	27.62	16.93	39.56	93.55	158.73
		K_L (L/mg)	0.040	0.112	0.064	0.055	0.021	0.021
		R^2	0.9967	0.9984	0.9991	0.9984	0.9838	0.9731
	Freundlich	K_F(mg/g (L/mg)$^{1/n}$)	6.84	12.93	4.90	18.16	28.87	65.17
		$1/n$	0.120	0.118	0.191	0.113	0.161	0.112
		R^2	0.9183	0.9369	0.7131	0.9417	0.9373	0.9216
	Temkin	A	52.7	556.6	3.3	431.3	25.8	104.2
		B	1734.0	1164.8	1134.7	832.5	297.4	203.6
		R^2	0.8768	0.9594	0.7488	0.944	0.9150	0.8090
308.15	Langmuir	q_{max} (mg/g)	17.04	31.06	38.37	58.48	96.90	161.29
		K_L (L/mg)	0.075	0.040	0.007	0.026	0.021	0.032
		R^2	0.9975	0.9875	0.9241	0.9935	0.9823	0.9895
	Freundlich	K_F(mg/g (L/mg)$^{1/n}$)	9.58	13.23	4.19	20.11	30.41	59.31
		$1/n$	0.086	0.125	0.297	0.144	0.158	0.138
		R^2	0.6826	0.9204	0.9187	0.9570	0.9438	0.9685
	Temkin	A	5256.4	301.7	0.2	32.9	19.5	31.6
		B	2313	1067	414.0	496.0	286.9	175.7
		R^2	0.6066	0.9051	0.8156	0.8819	0.9223	0.9152

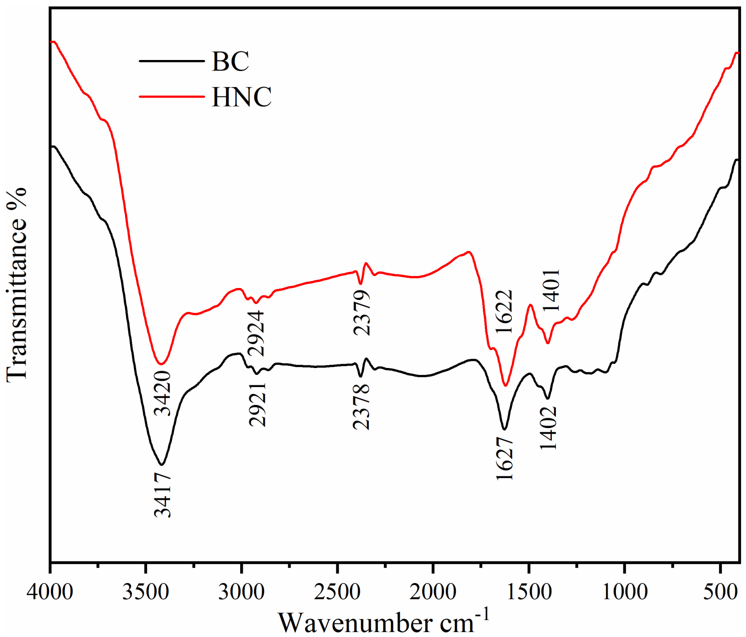

Fig.S1 FT-IR spectra of BC and HNC

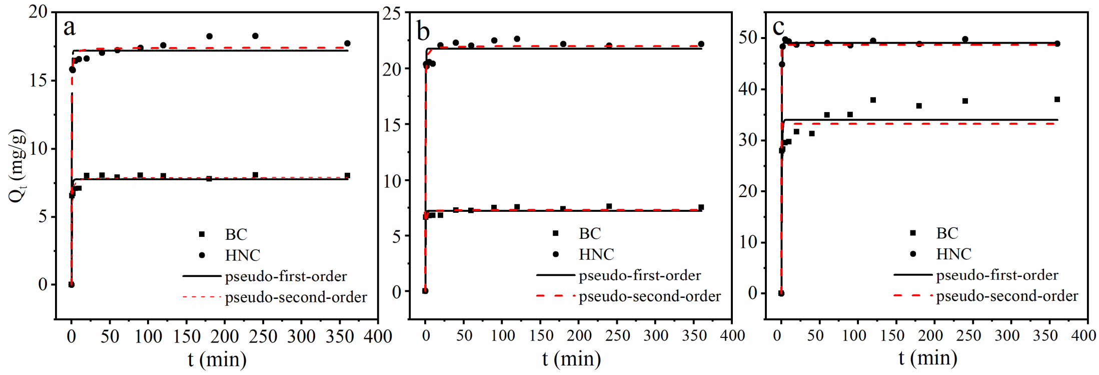

Fig.S2 Pseudo-first-order kinetic and pseudo-second-order kinetic fitting of Cu^{2+} (a), Cd^{2+} (b) and Pb^{2+} (c) adsorption by BC and HNC, dosage of 2 g/L for Cu^{2+} and Cd^{2+}, and 1 g/L for Pb^{2+}, pH 5.5, initial concentration of Cu^{2+}, Cd^{2+} and Pb^{2+} 50 mg/L, and temperature 298.15K
